# Supplementary material for: Cost-Efficient Detection of NTRK1/2/3 Gene Fusions: Single-Center Analysis of 8075 Tumor Samples
Source: Int J Mol Sci. 2023 Sep 17;24(18):14203. doi: 10.3390/ijms241814203 (PMC10531831; doi:10.3390/ijms241814203)
Supplement: Supplementary file 1 [file ijms-24-14203-s001.zip › ijms-2553600-supplementary.pdf]

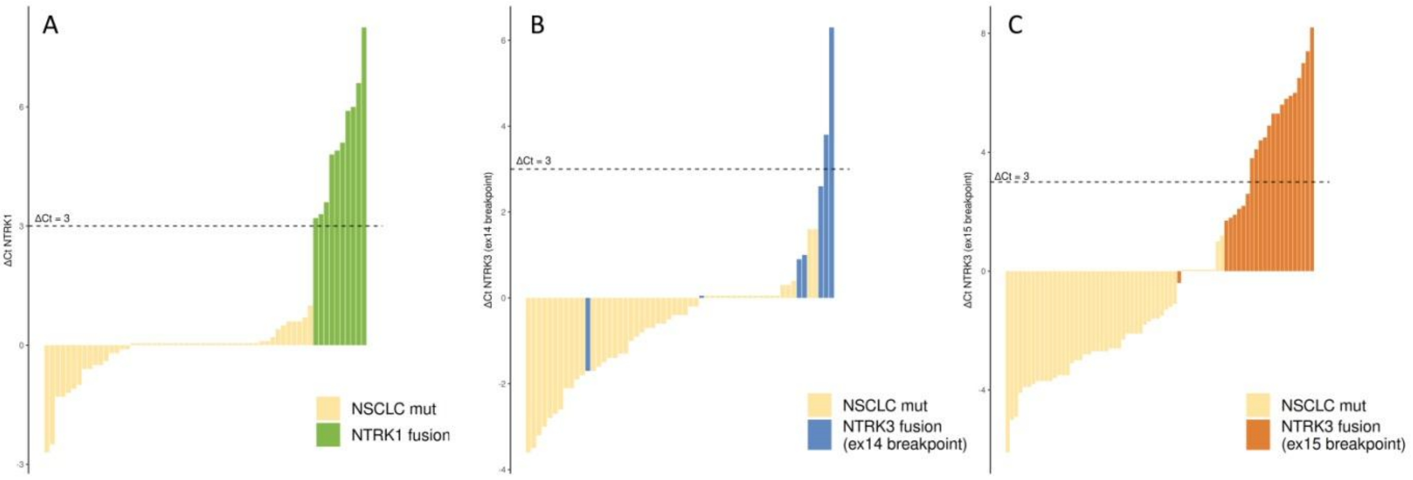

Figure S1. Distribution of delta Ct values obtained in unbalanced *NTRK* expression assays in *NTRK*-negative and *NTRK*-positive cases. (A) *NTRK1* 5′-3′ unbalanced expression assay. (B) *NTRK3* unbalanced breakpoint (ex13-14) expression assay. (C) *NTRK3* unbalanced breakpoint (ex14-15) expression assay. The same type of analysis was not performed for *NTRK2* due to low number of cases with *NTRK2* fusions. NSCLC mut – tumors with driver alterations affecting *EGFR*, *ALK*, *RET*, *ROS1* or *MET* genes (n = 50), which are highly unlikely to carry *NTRK* translocations due to mutually exclusive nature of these events.

Table S1. Histological types of salivary gland carcinomas and sarcomas

**Adult tumors**				
**Diagnosis**	**Histological type**	**Total cases, n**	**Cases with good-quality RNA, n**	**Cases with NTRK fusion, n**
Salivary gland carcinoma	adenocarcinoma, NOS	47	41	**2**
Salivary gland carcinoma	salivary duct carcinoma	18	17	**2**
Salivary gland carcinoma	adenoid cystic carcinoma	11	8	0
Salivary gland carcinoma	carcinoma ex pleomorphic adenoma	8	7	0
Salivary gland carcinoma	squamous cell carcinoma	5	4	0
Salivary gland carcinoma	epithelial-myoepithelial carcinoma	4	3	0
Salivary gland carcinoma	acinic cell carcinoma	2	2	0
Salivary gland carcinoma	mucoepidermoid carcinoma	2	2	0
Salivary gland carcinoma	secretory carcinoma (mammary analogue secretory carcinoma)	2	2	**1**
Salivary gland carcinoma	basal cell adenocarcinoma	1	1	0
Salivary gland carcinoma	carcinosarcoma	1	1	0
Salivary gland carcinoma	clear cell carcinoma	1	1	0
Sarcoma	sarcoma, NOS	29	28	0
Sarcoma	leiomyosarcoma	19	18	0
Sarcoma	liposarcoma	13	12	0
Sarcoma	Ewing sarcoma	10	8	0
Sarcoma	chondroblastic osteosarcoma	8	6	0
Sarcoma	malignant peripheral nerve sheath tumor (MPNST)	6	5	0
Sarcoma	synovial sarcoma	6	5	0
Sarcoma	endometrial stromal sarcoma	5	5	0
Sarcoma	rhabdomyosarcoma, NOS	4	3	0
Sarcoma	fibrosarcoma	3	3	0
Sarcoma	osteosarcoma	3	0	0
Sarcoma	undifferentiated pleomorphic sarcoma	3	2	0
Sarcoma	embryonal rhabdomyosarcoma	2	2	0
Sarcoma	myxofibrosarcoma (MFS)	2	2	0
Sarcoma	Ewing-like sarcoma	1	1	0
Sarcoma	alveolar rhabdomyosarcoma	1	1	0
Sarcoma	clear cell sarcoma	1	1	0

Sarcoma	hemangioendothelioma	1	1	0
Sarcoma	spindle cell sarcoma	1	1	0
**Pediatric tumors**				
Salivary gland carcinoma	adenocarcinoma, NOS	2	2	0
Salivary gland carcinoma	adenoid cystic carcinoma	1	1	0
Salivary gland carcinoma	epithelial-myoepithelial carcinoma	1	1	0
Sarcoma	Ewing sarcoma	8	4	0
Sarcoma	sarcoma, NOS	8	8	**2**
Sarcoma	alveolar rhabdomyosarcoma	6	6	0
Sarcoma	malignant peripheral nerve sheath tumor (MPNST)	5	5	**1**
Sarcoma	infantile fibrosarcoma	3	3	**2**
Sarcoma	rhabdomyosarcoma, NOS	3	2	0
Sarcoma	dermatofibrosarcoma protuberans (DFSP)	2	2	**1**
Sarcoma	embryonal rhabdomyosarcoma	2	2	0
Sarcoma	osteosarcoma	2	0	0
Sarcoma	synovial sarcoma	2	2	0
Sarcoma	angiosarcoma	1	1	0
Sarcoma	clear cell sarcoma	1	1	0
Sarcoma	hemangioendothelioma	1	1	**1**
Sarcoma	liposarcoma	1	1	0
Sarcoma	spindle cell rhabdomyosarcoma	1	1	0

Table S2. Primers and probes used for unbalanced NTRK expression testing

**SDHA**	
SDHA F	CCACTCGCTATTGCACACC
SDHA R	ATCCAAGGCAAAATACTCCAC
SDHA P	[R6G]CTGGTATCATATCGCAGAGACC[BHQ2]
**NTRK1**	
NTRK1 ex 3 F	GCCTTCCATTTCACTCCTCG
NTRK1 ex 4 R	GAGAGAGACTCCAGAGCGT
NTRK1 ex 3-4 P	[FAM]TCAGTCGCCTGAATCTCTCCTTCAAC[BHQ1]
NTRK1 ex 14 F	GCTCATGGTCTTTGAGTATATGCG
NTRK1 ex 15 R	AGCTTGGCATCAGGTCCATG
NTRK1 ex 14-15 P	[JOE]ATCGGAGGAAGCGGTTGAGGTC[BHQ1]
**NTRK2**	
NTRK2 ex 11 F	AATGAAATCCCTTCCACAGACG
NTRK2 ex 12 R	CCACCACAGACGCAATCAC
NTRK2 ex 11-12 P	[FAM]AACATCTCTCGGTCTATGCTGTGGT[BHQ1]
NTRK2 ex 15 F	GAATGCTATAACCTCTGTCCTGAG
NTRK2 ex 16 R	CACGGTGGAAGTCCTTGC
NTRK2 ex 15-16 P	[CY35]TTCAGGGTCTTCACTGCCACCAAGAT[BHQ2]
**NTRK3**	
NTRK3 ex 13 F	GGTTCTCTTCGTCATGATCAAC
NTRK3 ex 14 R	CCTCCTCACCACTGATGAC
NTRK3 ex 13-14 P	[FAM]TTTGGAATGAAGGGTCCCGTGGCT[BHQ1]
NTRK3 ex 14 F	CCAGTACTTCCGTCAGGGAC
NTRK3 ex 15 R	GTCTCTCCTCTTAATGTGCTGC
NTRK3 ex 14-15 P	[ROX]AACTGCCACAAGCCGGACACGTATG[BHQ2]
NTRK3 ex 16 F	AATACATGAAGCATGGAGACC
NTRK3 ex 17 R	CACAAGGATCATTGCATCTGG
NTRK3 ex 16-17 P	[JOE]CATGGGCCCTGAGGAACTTATT[BHQ1]

Table S3. Primers and probes used for variant-specific PCR

#	Breakpoint	Oligo name	Strand	Sequence	Fragment size
**NTRK1 variants**					
**LMNA::NTRK1**					
1	(L3;N10)	LMNA ex 3 F	Forward	ACTTCCAGAAGAACATCTACAGTG	75
		LMNA-NTRK1 ex 10 R	Reverse	GGTGTTTCGTCCTTCTTCTCC	
		LMNA-NTRK1 P1	Probe	[FAM]ACACTAACAGCACATCTGGAGACCCG[BHQ1]	
2	(L3;N11)	LMNA ex 3 F	Forward	ACTTCCAGAAGAACATCTACAGTG	69
		LMNA-NTRK1 ex 11 R	Reverse	GGAAGAGGCAGGCAAAGAC	
		LMNA-NTRK1 P2	Probe	[FAM]TCTCGGTGGCTGTGGGCCTG[BHQ1]	
3	(L3;N12)	LMNA ex 3 F	Forward	ACTTCCAGAAGAACATCTACAGTG	78
		LMNA-NTRK1 ex 12 R	Reverse	ATGAAATGCAGGGACATGGC	
		LMNA-NTRK1 P3	Probe	[FAM]CTGTGCTGGCTCCAGAGGATGGG[BHQ1]	
4	(L4;N10)	LMNA ex 4 F	Forward	GCTGGAGAAGACTTATTCTGCC	74
		LMNA-NTRK1 ex 10 R	Reverse	GGTGTTTCGTCCTTCTTCTCC	
		LMNA-NTRK1 P1	Probe	[FAM]ACACTAACAGCACATCTGGAGACCCG[BHQ1]	
5	(L4;N11)	LMNA ex 4 F	Forward	GCTGGAGAAGACTTATTCTGCC	68
		LMNA-NTRK1 ex 11 R	Reverse	GGAAGAGGCAGGCAAAGAC	
		LMNA-NTRK1 P2	Probe	[FAM]TCTCGGTGGCTGTGGGCCTG[BHQ1]	
6	(L4;N12)	LMNA ex 4 F	Forward	GCTGGAGAAGACTTATTCTGCC	77
		LMNA-NTRK1 ex 12 R	Reverse	ATGAAATGCAGGGACATGGC	
		LMNA-NTRK1 P3	Probe	[FAM]CTGTGCTGGCTCCAGAGGATGGG[BHQ1]	
7	(L8;N10)	LMNA ex 8 F	Forward	CCACCAAAGTTCACCCTGAAG	88
		LMNA-NTRK1 ex 10 R	Reverse	GGTGTTTCGTCCTTCTTCTCC	
		LMNA-NTRK1 P1	Probe	[FAM]ACACTAACAGCACATCTGGAGACCCG[BHQ1]	
8	(L8;N11)	LMNA ex 8 F	Forward	CCACCAAAGTTCACCCTGAAG	82
		LMNA-NTRK1 ex 11 R	Reverse	GGAAGAGGCAGGCAAAGAC	
		LMNA-NTRK1 P2	Probe	[FAM]TCTCGGTGGCTGTGGGCCTG[BHQ1]	
9	(L8;N12)	LMNA ex 8 F	Forward	CCACCAAAGTTCACCCTGAAG	91
		LMNA-NTRK1 ex 12 R	Reverse	ATGAAATGCAGGGACATGGC	
		LMNA-NTRK1 P3	Probe	[FAM]CTGTGCTGGCTCCAGAGGATGGG[BHQ1]	

10	(L10;N10)	LMNA ex 10 F	Forward	TGAGGATGGAGATGACCTGC	83
		LMNA-NTRK1 ex 10 R	Reverse	GGTGTTTCGTCCTTCTTCTCC	
		LMNA-NTRK1 P1	Probe	[FAM]ACACTAACAGCACATCTGGAGACCCG[BHQ1]	
11	(L10;N11)	LMNA ex 10 F	Forward	TGAGGATGGAGATGACCTGC	77
		LMNA-NTRK1 ex 11 R	Reverse	GGAAGAGGCAGGCAAAGAC	
		LMNA-NTRK1 P2	Probe	[FAM]TCTCGGTGGCTGTGGGCCTG[BHQ1]	
12	(L10;N12)	LMNA ex 10 F	Forward	TGAGGATGGAGATGACCTGC	86
		LMNA-NTRK1 ex 12 R	Reverse	ATGAAATGCAGGGACATGGC	
		LMNA-NTRK1 P3	Probe	[FAM]CTGTGCTGGCTCCAGAGGATGGG[BHQ1]	
13	(L11;N10)	LMNA ex 11 F	Forward	TACCTCCTGGGCAACTCC	82
		LMNA-NTRK1 ex 10 R	Reverse	GGTGTTTCGTCCTTCTTCTCC	
		LMNA-NTRK1 P1	Probe	[FAM]ACACTAACAGCACATCTGGAGACCCG[BHQ1]	
14	(L11;N11)	LMNA ex 11 F	Forward	TACCTCCTGGGCAACTCC	76
		LMNA-NTRK1 ex 11 R	Reverse	GGAAGAGGCAGGCAAAGAC	
		LMNA-NTRK1 P2	Probe	[FAM]TCTCGGTGGCTGTGGGCCTG[BHQ1]	
15	(L11;N12)	LMNA ex 11 F	Forward	TACCTCCTGGGCAACTCC	85
		LMNA-NTRK1 ex 12 R	Reverse	ATGAAATGCAGGGACATGGC	
		LMNA-NTRK1 P3	Probe	[FAM]CTGTGCTGGCTCCAGAGGATGGG[BHQ1]	
**BCAN::NTRK1**					
16	(B12;N10)	BCAN ex 12 F	Forward	CAACTACCACCTGTCCTACAC	86
		BCAN-NTRK1 ex 10 R	Reverse	GTGTTTCGTCCTTCTTCTCCA	
		BCAN-NTRK1 P	Probe	[FAM]CACTAACAGCACATCTGGAGACC[BHQ1]	
**IRF2BP2::NTRK1**					
17	(I1;N8)	IRF2BP2 ex 1 F	Forward	AGGCAGGTTGTTGGGTTTC	118
		IRF2BP2-NTRK1 ex 8 R	Reverse	ATCCACAGAGAAGGGGATGC	
		IRF2BP2-NTRK1 P	Probe	[FAM]ACACTAACAGCACATCTGGAGACCCG[BHQ1]	
18	(I1;N10)	IRF2BP2 ex 1 F	Forward	AGGCAGGTTGTTGGGTTTC	74
		IRF2BP2-NTRK1 ex 10 R	Reverse	GGTCTCCAGATGTGCTGTTAG	
		IRF2BP2-NTRK1 P	Probe	[FAM]ACACTAACAGCACATCTGGAGACCCG[BHQ1]	
**TPM3::NTRK1**					
19	(T7;N9)	TPM3 ex 7 F	Forward	CAAGATTCTTACTGATAAACTCAAGGAG	45
		TPM3-NTRK1 ex 9 R	Reverse	CACCGGCGAGAAGGAGA	
		TPM3-NTRK1 P1	Probe	[JOE]CTTCCAGGTCATCAATTGTCTTTTCCAGC[BHQ1]	
20	(T7;N10)	TPM3 ex 7 F	Forward	CAAGATTCTTACTGATAAACTCAAGGAG	76

		TPM3-NTRK1 ex 10 R	Reverse	GTGTTTCGTCCTTCTTCTCCA	
		TPM3-NTRK1 P3	Probe	[FAM]CACTAACAGCACATCTGGAGACC[BHQ1]	
21	(T7;N12)	TPM3 ex 7 F	Forward	CAAGATTCTTACTGATAAACTCAAGGAG	80
		TPM3-NTRK1 ex 12 R	Reverse	ATGAAATGCAGGGACATGGC	
		TPM3-NTRK1 P4	Probe	[FAM]CTGTGCTGGCTCCAGAGGATGGG[BHQ1]	
22	(T8;N9)	TPM3 ex 8 F	Forward	GAGATCGGTAGCCAAGCTG	61
		TPM3-NTRK1 ex 9 R	Reverse	CACCGGCGAGAAGGAGA	
		TPM3-NTRK1 P1	Probe	[JOE]CTTCCAGGTCATCAATTGTCTTTTCCAGC[BHQ1]	
23	(T8;N10)	TPM3 ex 8 F	Forward	GAGATCGGTAGCCAAGCTG	92
		TPM3-NTRK1 ex 10 R	Reverse	GTGTTTCGTCCTTCTTCTCCA	
		TPM3-NTRK1 P3	Probe	[FAM]CACTAACAGCACATCTGGAGACC[BHQ1]	
24	(T8;N12)	TPM3 ex 8 F	Forward	GAGATCGGTAGCCAAGCTG	96
		TPM3-NTRK1 ex 12 R	Reverse	ATGAAATGCAGGGACATGGC	
		TPM3-NTRK1 P4	Probe	[FAM]CTGTGCTGGCTCCAGAGGATGGG[BHQ1]	
25	(T9;N9)*	TPM3 ex 9 F	Forward	CTGGACCACGCCCTCAATG	98
		TPM3-NTRK1 ex 10 R	Reverse	GTGTTTCGTCCTTCTTCTCCA	
		TPM3-NTRK1 P3	Probe	[FAM]CACTAACAGCACATCTGGAGACC[BHQ1]	
26	(T9;N10)*	TPM3 ex 9 F	Forward	CTGGACCACGCCCTCAATG	80
		TPM3-NTRK1 ex 10 R	Reverse	GTGTTTCGTCCTTCTTCTCCA	
		TPM3-NTRK1 P3	Probe	[FAM]CACTAACAGCACATCTGGAGACC[BHQ1]	
27	(T9;N12)	TPM3 ex 9 F	Forward	CTGGACCACGCCCTCAATG	84
		TPM3-NTRK1 ex 12 R	Reverse	ATGAAATGCAGGGACATGGC	
		TPM3-NTRK1 P4	Probe	[FAM]CTGTGCTGGCTCCAGAGGATGGG[BHQ1]	
28	(T10;N9)	TPM3 ex 10 F	Forward	ATGGATTTAGTACAGGTTACTCAGG	110
		TPM3-NTRK1 ex 9 R	Reverse	CACCGGCGAGAAGGAGA	
		TPM3-NTRK1 P2	Probe	[FAM]AAGGAATTTAATCTTGTTCAGCTTGAGGAG[BHQ1]	
29	(T10;N10)	TPM3 ex 10 F	Forward	ATGGATTTAGTACAGGTTACTCAGG	141
		TPM3-NTRK1 ex 10 R	Reverse	GTGTTTCGTCCTTCTTCTCCA	
		TPM3-NTRK1 P2	Probe	[FAM]AAGGAATTTAATCTTGTTCAGCTTGAGGAG[BHQ1]	
30	(T10;N12)	TPM3 ex 10 F	Forward	ATGGATTTAGTACAGGTTACTCAGG	145
		TPM3-NTRK1 ex 12 R	Reverse	ATGAAATGCAGGGACATGGC	
		TPM3-NTRK1 P2	Probe	[FAM]AAGGAATTTAATCTTGTTCAGCTTGAGGAG[BHQ1]	
**NTRK2 variants**					
**BCR::NTRK2**					

31	(B1;N17)	BCR ex 1 F	Forward	GCCCAACGATGGCGAG	80
		BCR-NTRK2 ex17 R	Reverse	GGGTTGCCCTCAGCC	
		BCR-NTRK2 P	Probe	[FAM]ATGGAGACGCAGGGCACACGG[BHQ1]	
**SQSTM1::NTRK2**					
32	(S5;N16)	SQSTM1 ex 5 F	Forward	TCCGAGTGTGAATTTCCTGAAG	108
		SQSTM1-NTRK2 ex 16 R	Reverse	CACGGTGGAAGTCCTTGC	
		SQSTM1-NTRK2 P	Probe	[FAM]ACCCTGAAGGATGCCAGTGACAAT[BHQ1]	
33	(S6;N16)	SQSTM1 ex 6 F	Forward	TCGCCTTGGAGTCCGAG	81
		SQSTM1-NTRK2 ex 16 R	Reverse	CACGGTGGAAGTCCTTGC	
		SQSTM1-NTRK2 P	Probe	[FAM]ACCCTGAAGGATGCCAGTGACAAT[BHQ1]	
**NTRK3 variants**					
**ETV6::NTRK3**					
34	(E4;N13)	ETV6 ex 4 F	Forward	CATTCTTCCACCCTGGAAACTC	92
		ETV6-NTRK3 ex 13 R	Reverse	GCAAGTCCAACTGCTATGGATAC	
		ETV6-NTRK3 P1	Probe	[FAM]TTCTGATGCAGTATGACCTCCGGCTGT[BHQ1]	
35	(E4;N14)	ETV6 ex 4 F	Forward	CATTCTTCCACCCTGGAAACTC	90
		ETV6-NTRK3 ex 14 R	Reverse	CACTGATGACAGCCACGG	
		ETV6-NTRK3 P1	Probe	[FAM]TTCTGATGCAGTATGACCTCCGGCTGT[BHQ1]	
36	(E4;N15)	ETV6 ex 4 F	Forward	CATTCTTCCACCCTGGAAACTC	123
		ETV6-NTRK3 ex 15 R	Reverse	CCTCACCCAGTTCTCGCTT	
		ETV6-NTRK3 P1	Probe	[FAM]TTCTGATGCAGTATGACCTCCGGCTGT[BHQ1]	
37	(E5;N13)	ETV6 ex 5 F	Forward	CTGGCTTACATGAACCACATCA	99
		ETV6-NTRK3 ex 13 R	Reverse	GCAAGTCCAACTGCTATGGATAC	
		ETV6-NTRK3 P2	Probe	[FAM]TGCTATTCTCCCAATGGGCATGGCGT[BHQ1]	
38	(E5;N14)	ETV6 ex 5 F	Forward	CTGGCTTACATGAACCACATCA	97
		ETV6-NTRK3 ex 14 R	Reverse	CACTGATGACAGCCACGG	
		ETV6-NTRK3 P2	Probe	[FAM]TGCTATTCTCCCAATGGGCATGGCGT[BHQ1]	
39	(E5;N15)	ETV6 ex 5 F	Forward	CTGGCTTACATGAACCACATCA	130
		ETV6-NTRK3 ex 15 R	Reverse	CCTCACCCAGTTCTCGCTT	
		ETV6-NTRK3 P2	Probe	[FAM]TGCTATTCTCCCAATGGGCATGGCGT[BHQ1]	
40	(E6;N13)	ETV6 ex 6 F	Forward	CCGGATAGTGGATCCCAAC	72
		ETV6-NTRK3 ex 13 R	Reverse	GCAAGTCCAACTGCTATGGATAC	
		ETV6-NTRK3 P3	Probe	[FAM]TTATGGTTTCCCCACAGTCGAGCCAGT[BHQ1]	
41	(E6;N14)	ETV6 ex 6 F	Forward	CCGGATAGTGGATCCCAAC	70

		ETV6-NTRK3 ex 14 R	Reverse	CACTGATGACAGCCACGG	
		ETV6-NTRK3 P3	Probe	[FAM]TTATGGTTTCCCCACAGTCGAGCCAGT[BHQ1]	
42	(E6;N15)	ETV6 ex 6 F	Forward	CCGGATAGTGGATCCCAAC	103
		ETV6-NTRK3 ex 15 R	Reverse	CCTCACCCAGTTCTCGCTT	
		ETV6-NTRK3 P3	Probe	[FAM]TTATGGTTTCCCCACAGTCGAGCCAGT[BHQ1]	
**EML4::NTRK3**					
43	(E2;N13)	EML4 ex 2 F	Forward	CTTTGGCTGATGTTTTGAGGC	101
		EML4-NTRK3 ex 13 R	Reverse	GCAAGTCCAACTGCTATGGATAC	
		EML4-NTRK3 P	Probe	[FAM]TGAGACTGATTTTTTCACTGAGGCCAC[BHQ1]	
44	(E2;N14)	EML4 ex 2 F	Forward	CTTTGGCTGATGTTTTGAGGC	99
		EML4-NTRK3 ex 14 R	Reverse	CACTGATGACAGCCACGG	
		EML4-NTRK3 P	Probe	[FAM]TGAGACTGATTTTTTCACTGAGGCCAC[BHQ1]	

* fragments TPM3ex9/NTRK1ex9 and TPM3ex9/NTRK1ex10 are produced by the same primer pair and can be differentiated by fragment size

Table S4. List of transcripts utilized for primer design

Gene	Transcript
NTRK1	NTRK1-206 ENST00000524377.7
NTRK2	NTRK2-201 ENST00000277120.8
NTRK3	NTRK3-205 ENST00000394480.6
LMNA	LMNA-205 ENST00000368300.9
BCAN	BCAN-201 ENST00000329117.10
IRF2BP2	IRF2BP2-201 ENST00000366609.4
TPM3	TPM3-224 ENST00000651641.1
BCR	BCR-201 ENST00000305877.13
SQSTM1	SQSTM1-202 ENST00000389805.9
ETV6	ETV6-202 ENST00000396373.9
EML4	EML4-201 ENST00000318522.10
